# Mitochondrial Protection and Anti-aging Activity of *Astragalus* Polysaccharides and Their Potential Mechanism

**DOI:** 10.3390/ijms13021747

**Published:** 2012-02-07

**Authors:** Xing-Tai Li, Ya-Kui Zhang, Hai-Xue Kuang, Feng-Xin Jin, De-Wen Liu, Ming-Bo Gao, Ze Liu, Xiao-Juan Xin

**Affiliations:** 1 College of Life Science, Dalian Nationalities University, No.18 Liaohe West Road, Dalian Economic & Technical Development Zone, Dalian 116600, China; E-Mails: lily@dlnu.edu.cn (M.-B.G.); liuze@dlnu.edu.cn (Z.L.); 2 Daxing’anling Academy of Agriculture and Forestry Sciences, Jiagedaqi 165000, China; E-Mails: zhangyk02@yahoo.com.cn (Y.-K.Z.); lksjfx@126.com (F.-X.J.); ldwen817@yahoo.com.cn (D.-W.L.); xinxiaojuan323@163.com (X.-J.X.); 3 College of Pharmacy, Heilongjiang University of Traditional Chinese Medicine, Harbin 150040, China; E-Mail: hxkuang@hotmail.com

**Keywords:** *Astragalus* polysaccharides, mitochondria, anti-aging, antioxidant, reactive oxygen species, mitochondrial permeability transition

## Abstract

The current study was performed to investigate mitochondrial protection and anti-aging activity of *Astragalus* polysaccharides (APS) and the potential underlying mechanism. Lipid peroxidation of liver and brain mitochondria was induced by Fe^2+^–Vit C *in vitro*. Thiobarbituric acid (TBA) colorimetry was used to measure the content of thiobarbituric acid reactive substances (TBARS). Mouse liver mitochondrial permeability transition (PT) was induced by calcium overload *in vitro* and spectrophotometry was used to measure it. The scavenging activities of APS on superoxide anion (O_2_^•−^) and hydroxyl radical (•OH), which were produced by reduced nicotinamide adenine dinucleotide (NADH)—N-Methylphenazonium methyl sulfate (PMS) and hydrogen peroxide (H_2_O_2_)–Fe^2+^ system respectively, were measured by 4-nitrobluetetrazolium chloride (NBT) reduction and Fenton reaction colorimetry respectively. The Na_2_S_2_O_3_ titration method was used to measure the scavenging activities of APS on H_2_O_2_. APS could inhibit TBARS production, protect mitochondria from PT, and scavenge O_2_^•−^, •OH and H_2_O_2_ significantly in a concentration-dependent manner respectively. The back of the neck of mice was injected subcutaneously with D-galactose to induce aging at a dose of 100 mg/kg/d for seven weeks. Moreover, the activities of catalase (CAT), surperoxide dismutase (SOD) and glutathione peroxidase (GPx) and anti-hydroxyl radical which were assayed by using commercial monitoring kits were increased significantly *in vivo* by APS. According to this research, APS protects mitochondria by scavenging reactive oxygen species (ROS), inhibiting mitochondrial PT and increasing the activities of antioxidases. Therefore, APS has the effect of promoting health.

## 1. Introduction

The free radical theory of aging is based on the works of Gerschman and Harman, and when focused in mitochondria emerged as the mitochondrial hypothesis of aging [1,2]. It holds that during aging, an increase in reactive oxygen species (ROS) in mitochondria causes mutations in the mtDNA and damages mitochondrial components, resulting in senescence [3]. Mitochondria are considered the pacemakers of tissue aging due to the continuous production of free radicals, oxygen, and nitrogen free radicals and related reactive species, and to the selective oxidative damage that leads to mitochondrial dysfunction [4]. Mitochondria are the driving force behind life, as mitochondrial oxidative phosphorylation provides the main source of energy in the cell. In addition to energy production, mitochondria play a crucial role in mediating amino acid biosynthesis, fatty acid oxidation, steroid metabolism, intermediate metabolic pathways, calcium homeostasis, and free radical scavenging [5]. Mitochondria are a major source of ROS, which are a byproduct of mitochondrial electron transfer activity. Molecular oxygen (O_2_) is highly electrophilic and superoxide production occurs when O_2_ captures an electron from Complex I or from the ubisemiquinone located in Complex III. Normally, superoxide is detoxified by the combined activity of the mitochondrial antioxidant enzymes manganese surperoxide dismutase (MnSOD), catalase (CAT) and glutathione peroxidase (GPx) [6]. Superoxide anion (O_2_^•−^) is the precursor of most ROS and is a mediator in oxidative chain reactions. Dismutation of O_2_^•−^, either spontaneously or through a catalytic reaction by surperoxide dismutase (SOD), produces hydrogen peroxide (H_2_O_2_), which in turn may be fully reduced to water by CAT and GPx or partially reduced to a hydroxyl radical (•OH), one of the strongest oxidants in nature [7]. •OH may be re-reduced by O_2_^•−^ and may propagate the entire ROS process [8]. As a consequence of their biological functions, mitochondria are always exposed to ROS production and have a complex antioxidant defense system to counteract it. Oxidative stress occurs when the homeostatic balance between oxidant and antioxidant capacities in a determined biological system is disturbed [9]. Thus, under conditions of oxidative stress, mitochondria may suffer from oxidative damage to their biomolecules. Since the removal and repair of altered structures may not be completely efficient, the oxidizing products might accumulate in this organelle.

Astragali Radix is derived from the dried roots of *Astragalus membranaceus* (Fisch.) Bunge or *A. membranaceus* (Fisch.) Bunge var. *mongholicus* (Bunge) Hsiao (AM, Huang-qi in Chinese)*,* is one of the most frequently used Qi-invigorating herbal medicines in traditional Chinese medicine for millennia. AM is often used in formulas for deficiency of Qi (vital energy) characterized by limb weakness, fatigue, lack of appetite, and dizziness. It is considered that AM is the most popular for Qi-tonifying herbal medicines and is often used as an antiperspirant, an immunostimulant, a diuretic, and a supplementary medicine during cancer therapy [10]. AM has various bioactivities, such as anti-aging [11], hepatoprotective, antibacterial, inducing cancer cell apoptosis [12], and preventing apoptosis in cultured neonatal cardiomyocytes [13]. AM inhibited mitochondrial oxygen consumption and malondialdehyde (MDA) production [14]. Recently, most of the constituents including triterpene saponins, isoflavonoids, and polysaccharides were isolated from AM, and their bioactive effects were also investigated [15,16]. As an important bioactive component of AM, *Astragalus* polysaccharides (APS) have immunoregulatory, antiviral, hypoglycemic, antioxidant, and antitumor properties [17–20]. Therefore, APS have received a great deal of attention [21] and have been applied in the treatment of many diseases, including tumor and infectious diseases, in Chinese medicine [22]. APS therapy ameliorated vacuolar degeneration of mitochondria and fragmentation of mitochondrial cristae of hepatocytes in insulin-resistance mice, which indicates the mitochondrial dysfunction coupled with the increased metabolic stress and the protective effect of APS [19].

However, there has been no report on how mitochondria were protected and the mechanism of protective effect on mitochondrial injury by APS so far. As the ‘hubs’ for cellular metabolism, mitochondria are crucial for both life and death of eukaryotic cells, and are the main switch of cell apoptosis. In the present study, the mechanism underlying mitochondrial protection and anti-aging activities of APS were investigated and evaluated by the inhibition of mitochondrial permeability transition (PT), the scavenging abilities on ROS and the effects on activities of the antioxidases.

## 2. Results and Discussion

### 2.1. Lipid Peroxidation Prevented in Liver and Brain Mitochondria *in Vitro*

Peroxidation of membrane lipids has been suggested to be one of the major causes of decreased mitochondrial membrane function [23]. Oxidative damage was studied by measuring lipid peroxidation (LPO) products such as thiobarbituric acid reactive substances (TBARS). The TBARS level, used as an auto-oxidation index of LPO in tissues, is used to screen the antioxidants. Pro-oxidants and ROS result in LPO in mitochondria [24]. It is widely accepted that LPO increases with age [25]. In fact, peroxidation alters the structure of membrane lipids, which can disrupt the structural organization of the lipid double layer, altering membrane fluidity and permeability. A significant increase in the peroxidation products has been reported in rat liver mitochondria [26]. Also, in mice skeletal muscle mitochondria, Faist *et al.* have demonstrated an increased mitochondrial formation of TBARS with age [27]. Unsaturated lipids in liver tissue are very susceptible to peroxidation when they are exposed to pro-oxidative metal ions such as Fe^2+^. It has long been thought that Fe^2+^ is the most likely active species, producing oxidants through interaction of Fe^2+^ with oxygen. In the current investigation we have incubated the mouse liver and brain mitochondria with or without the presence of Fe^2+^, and examined their effects on mitochondria by measuring the absorbance at 532 nm. The current results show that formation of TBARS in mitochondria, which was enhanced significantly following treatment with Fe^2+^–Vitamin C, was inhibited in a concentration-dependent manner in presence of APS (Table 1), which indicates that APS possess the antioxidant activity.

### 2.2. Inhibition of Liver Mitochondrial Permeability Transition

Permeability and fluidity of mitochondrial membrane are prerequisites for maintaining mitochondrial functions; the status of the mitochondrial membrane potential, which is an estimate of the electrochemical gradient across the inner mitochondrial membrane, and thus the mitochondrial permeability transition pore (MPTP) opening. ROS can stimulate the opening of MPTP [28]. Mitochondrial PT is a sensitive index for surveying permeability of the membrane and assessing mitochondrial functions. High levels of pro-oxidants produced by mitochondria can induce apoptosis by changing cellular redox status, depleting reduced glutathione (GSH) [29]. Pro-oxidants and ROS result in opening of MPTP in mitochondria [24]. Onset of the permeability transition was monitored from the changes of absorbance at 540 nm, which reflect mitochondrial permeabilization to sucrose. Mitochondria were challenged with a Ca^2+^ load of 150 μM, which caused a detectable permeability transition. After Ca^2+^ accumulation ruthenium red (RR) was added to prevent Ca^2+^ redistribution [30], rapid and large amplitude mitochondrial swelling was induced by Ca^2+^, indicating that the swelling was because of the opening of the MPTP, and the effects of Ca^2+^ were blocked completely by 0.5 μM RR and partially blocked by 0.3 μM RR in this study. LPO can lead to increase of membrane permeability thus, mitochondrial swelling is initiated. APS inhibited Ca^2+^ induced mitochondrial PT significantly and the inhibitory potency was stronger when the incubation time was longer and concentration of APS was higher. No significant difference was observed between APS (64 mg/L) and the normal (Table 2). The inhibition of mitochondrial PT by APS was closely related to its scavenging activity on ROS and the inhibition on LPO, which indicates that maybe APS protect mitochondria by scavenging ROS and antioxidation.

### 2.3. Scavenging Activity of APS on Superoxide Anion, Hydroxyl Radicals and Hydrogen Peroxide

ROS are chemically reactive molecules derived from oxygen. There is increasing evidence that accumulation of ROS in biological system causes oxidative damage to tissue that affects cellular integrity and functions. Oxidative damage caused by ROS has been frequently proposed to be associated with the pathogenesis of various diseases and aging [31]. The mitochondrial electron transport chain (ETC) consumes more than 90% of the oxygen taken up by the cell, and up to 5% of that is converted into O_2_^•−^ even during a normal physiological state [32]. Superoxide anion, as the precursor of the more ROS including •OH and H_2_O_2_, is very harmful to the cellular components in a biological system. The primary ROS generated in the mitochondria is O_2_^•−^ which is then converted to H_2_O_2_ by spontaneous dismutation or by SOD [33]. Although H_2_O_2_ is not very reactive, its high penetrability of cellular membrane leads to •OH formation when it reacts with ferrous ion or O_2_^•−^. Hydroxyl radical is very reactive and can be generated through the Fenton reaction. Superoxide anion was generated by non-enzymatic reduced nicotinamide adenine dinucleotide/N-Methylphenazonium methyl sulfate (NADH/PMS) system in the present study. The absorbance (A) value at 560 nm of APS group decreased significantly compared with the control group. The current studies showed that APS scavenged O_2_^•−^, H_2_O_2_ and •OH concentration-dependently (Table 3 and 4). The abilities of APS and the reference compound [butylated hydroxytoluene (BHT) and vitamin C] to quench the three kinds of ROS are reflected in Tables 3 and 4. Scavenging rate (SR%) of APS (64 mg/L) for O_2_^•−^ and •OH are 60.64% and 85.29% respectively.

### 2.4. Improvement in the Activities of CAT, SOD, GPx and Anti-hydroxyl Radical

Vitamin E is the major lipid-soluble chain-breaking antioxidant in mammals and plays an important role in normal development and physiology [34]. Studies have shown that tocopherols scavenge and quench various reactive oxygen species and lipid oxidation by-products, which would otherwise propagate LPO chain reactions in membranes [35]. The standard antioxidant compound α**-**tocopherol, the most biologically active form of vitamin E [36], is a primary antioxidant functioning by terminating free-radical chain reactions by donating hydrogen or electrons to free radicals and converting them to more stable products [37]. α**-**Tocopherol has a potency to induce the increase in free radical-scavenging enzyme activities [38,39]. The lipophilic radical scavenger α-tocopherol, present in mitochondrial membranes, also has a role in interfering with the propagation of free radical-mediated chain reactions, thereby protecting membrane lipids from peroxidation [40]. CAT, SOD, GPx and anti-hydroxyl radical activities in liver homogenate of model group mice were decreased significantly *versus* normal group, the oral supplementation of vitamin E was found to increase the activities of CAT, SOD, GPx and anti-hydroxyl radical in this study. The ability of APS2 (200 mg/kg/d) on increasing CAT, SOD, GPx and anti-hydroxyl radical activities is vitamin E (100 mg/kg/d) comparable. Comparisons with model mice, the activities of CAT, SOD, GPx and anti-hydroxyl radical in APS3 group (300 mg/kg/d) were significantly increased, almost reaching normal levels (*P* > 0.05). APS increased CAT, SOD, GPx and anti-hydroxyl radical activities in a dose-dependent manner (Table 5). These results demonstrated that APS could enhance activities of the antioxidases and the ability of scavenging •OH *in vivo*.

Aging is a complex phenomenon, a sum total of changes that occur in a living organism with the passage of time and lead to decreasing ability to survive stress, increasing functional impairment and growing probability of death [41]. ROS are considered to be important causative factors in the aging process [42]. The processes that delay and/or reverse visible signs of aging are termed as anti-aging. Mitochondria are constantly exposed to the danger of ROS-induced oxidative injury. LPO is known to damage the mitochondrial membrane [43]. Approximately 1%–5% of the oxygen consumed by mitochondria in human cells is converted to ROS, including O_2_^•−^, H_2_O_2_, and •OH [44]. It has been shown that the amount of lipid peroxides in mitochondria increases with age [45]. Mitochondrial ROS production and oxidative damage in tissue cells are increased during aging. Cellular antioxidant systems have been traditionally divided into two categories: enzymatic and nonenzymatic. Primary antioxidant enzymes include SOD, GPx and CAT. Non-enzymatic antioxidants, like vitamin E (α-tocopherol) directly scavenge superoxide and •OH, as well as singlet oxygen [46]. GPx, which is probably the best studied mitochondrial antioxidant enzyme, plays an important role in the decomposition of H_2_O_2_ produced in mitochondria. GPx catalyzes H_2_O_2_ and ROOH reduction by GSH [H_2_O_2_ (ROOH) + 2 GSH => GSSG + 2H_2_O (ROH+ H_2_O)] and is the unique enzyme that uses H_2_O_2_ in the mitochondria of most mammalian organs [47]. In fact, GPx activity seems to exceed that of any competing H_2_O_2_ scavenger in mitochondria. Despite mostly present in the peroxisomes, CAT might also play a role in the decomposition of mitochondrial H_2_O_2_ to H_2_O [48]. The recent work by Schriner *et al.* clearly demonstrates that mitochondrially targeted CAT decreases free radicals (mainly H_2_O_2_), leads to reduced mitochondrial oxidative damage, and increases the lifespan of CAT transgenic mice [49], suggesting that overexpressed CAT in mitochondria decreases ROS and boosts the functioning of mitochondria.

## 3. Materials and Methods

### 3.1. Plant Materials and Animals

Astragali Radix was collected in the primitive forest of Daxing’anling region in Heilongjiang Province of China, which was authenticated by professor Haixue Kuang as the roots of *Astragalus membranaceus* (Fisch.) Bunge var. *mongholicus* (Bunge) Hsiao (AM), and its voucher specimen was deposited in the Herbarium of Chinese Herbal Medicines, College of Pharmacy, Heilongjiang University of Traditional Chinese Medicine.

Male BALB/c mice (Certificate No. SCXKGGD2004-0017), weighing 22 ± 2.0 g, were purchased from the Experimental Animal Center, Dalian Medical University. All mice were cared according to the Guiding Principles in the Care and Use of Animals. The experiment was approved by Animal Care Committee of Dalian Medical University (China) in accordance with the Chinese Council on Animal Care Guidelines. Rodent laboratory chow and tap water were available *ad libitum* during the period.

Animal groups of *in vivo* experiments: Sixty mice were randomly assigned into 6 groups (Normal, Model, Vit E and APS1, 2, 3. n = 10 for each group). APS (100, 200, 300 mg/kg/d) was administered by oral gavage to mice in APS1, 2, 3 group respectively. Vitamin E (100 mg/kg/d) was administered to mice in Vit E group and an equivalent volume of normal saline to mice in Normal and Model group. All the mice except the Normal group were administered D-galactose (100 mg/kg/d) subcutaneously and the Normal group mouse an equivalent volume of normal saline. Each index was determined 7 weeks later.

### 3.2. Chemicals

Coomassie brilliant blue G-250 (CBBG-250) and N-methylphenazonium methyl sulfate (PMS) were purchased from Fluka (Bushs SG, Switzerland). Bovine serum albumin (BSA), 4-nitrobluetetrazolium chloride (NBT) and NADH were from Boehringer Mannheim Corp. (Indianapolis, IN, USA). 2-Thiobarbituric acid (TBA) and 1, 1, 3, 3-tetraethoxypropane (TEP) were from Sigma Chemical (St Louis, MO, USA). HEPES was from Merck (Darmstadt, Germany). Tris was from Gibco BRL (Grand Island, NY, USA). 3-(*N*-Morpholino) propanesulfonic acid (MOPS) was from Solarbio (Beijing, China). Ruthenium red (RR) was from Alfa Aesar (Heysham, Lancashire, United Kingdom). Commercial CAT, SOD, GPx and anti-hydroxyl radical monitoring kits were from Nanjing Jiancheng Bioengineering Institute (Nanjing, China). The name of commercial kits used for determination of CAT, SOD, GPx and ant-hydroxyl radical activities are Reagent of Detection for Catalase, Surperoxide Dismutase, Glutathione Peroxidase and Reactive Oxygen Species respectively. Vitamin E (Vit E) was from Shanghai Xinyi Pharmaceutical Factory (Shanghai, China). D-galactose was from Shanghai Second Reagent Factory (Shanghai, China). All other chemicals and solvents used in the study were of analytical grade made in China.

### 3.3. Preparation of the Astragalus Polysaccharides

The collected AM was washed, dried, pulverized, then immersed in distilled water (the ratio of AM and distilled water was 1:15) for 24 h and extracted thrice with distilled water for 1 h each in a boiling water bath. The filtrate was collected after filtration with gauze, mixed and condensed to 1 g crude drug/mL by rotary evaporation. Sevage reagents (ratio of chloroform and n-butanol was 4:1) were used to remove all protein constituents. The resultant liquor was precipitated with 3 times volume of 95% ethanol. Precipitation of polysaccharides proceeded at 4 °C for 24 h and the precipitate which was collected by centrifugation at 5000 × g for 10 min was dissolved in distilled water, ethanol was added to final concentration of 25% to settle. The precipitate was discarded and the supernatant was added with 95% ethanol to final concentration of 75% and stood at 4 °C for 24 h after centrifugation at 5000 × g for 10 min. The resultant precipitate was washed with 95% ethanol and water-free ethanol respectively after suction and lyophilized *in vacuo*. The polysaccharides content (91.6%) in extracts was determined using the phenol-sulfuric acid method [50].

### 3.4. Isolation of Mitochondria

Mitochondria were isolated by differential centrifugation using a modified protocol of Fink *et al*. [51]. Mice were dislocated, the livers and brains were excised immediately, placed in precooled normal saline to wash the blood on the surface, then they were placed in an ice-cold isolation medium (containing 0.25 M sucrose, 0.5 mM EDTA and 3 mM HEPES, pH 7.4) and were homogenized with a motor-driven Teflon pestle in wet ice. Following homogenization, samples were centrifuged at 1000 × g for 10 min. A Beckman JA-25.50 rotor and Beckman Coulter Avanti J-E centrifuge were used in this and all other centrifugation steps at 4 °C. Supernatants were removed and centrifuged at 10,000 × g for 10 min. The pellets were washed twice in the isolation medium, and respun at 10,000 × g for 10 min each. After the final wash, mitochondria were resuspended in the same medium and stored in ice until use. Protein determinations were carried out by Bradford method using BSA as a standard [52].

### 3.5. Measurement of Thiobarbituric Acid Reactive Substances

The contents of TBARS in the liver and brain mitochondria were measured according to the method of Chen *et al.* with slight modification [53]. Mitochondrial protein (0.5 mg) in each tube was incubating with graded concentrations (2–32 mg/L) of APS (the normal and model group were excluded) at 37 °C for 5 min, then FeSO_4_ (0.25 mM, the normal group wasn’t added) and vitamin C (0.6 mM) were added. PBS (0.1 M, pH 7.4) was added to 2 mL (Mitochondria and APS were not added to the blank reference tube). After incubating the mixture in a vibratory incubator at 37 °C for 30 min, 0.5 mL of 20% trichloroacetic acid was added to end the reaction. Two milliliters supernatant was transferred to another tube after centrifugation (9000×g) for 10 min, to which 1.0 mL of 0.67% TBA was added and then heated in a boiling water bath for 10 min. After cooling with tap water, the absorbance at 532 nm was determined on an UV-Visible spectrophotometer and the blank reference tube was used to zero. TBARS contents (C) was measured by linear regression analysis of an aliquot using TEP as an external standard. The TBARS inhibition rate (IR%) was calculated according to the following equation:

$$\text{IR }\%=[(C_{\text{model}}-C_{\text{APS}})/(C_{\text{model}}-C_{\text{normal}})]\times 100$$

### 3.6. Evaluation of Mitochondrial Permeability Transition

Liver mitochondria were isolated and resuspended (0.25 mg of protein/mL) in an incubation medium (250 mM sucrose, 1 mM P_i_-Tris, 10 mM Tris-MOPS, 5 mM glutamate-Tris, 2.5 mM malate-Tris, pH 7.4, 25 °C). 150 μM Ca^2+^ was added followed by APS or ruthenium red (0.3 or 0.5 μM) (the model group was excluded). Experiments were started by the addition of 0.5 mg of mitochondria. The final volume was 2 mL. Mitochondrial PT was monitored as the absorbance (A) decrease of the mitochondrial suspension at 540 nm at 0, 2, 5, 10, 15, and 30 min [54,55].

### 3.7. Assay of Superoxide Anion Scavenging Activity

Superoxide anion was generated in NADH/PMS system and was measured by the nitrobluetetrazolium (NBT) reduction method that superoxide radicals reduce NBT into a purple-colored formazan [56]. The 3-mL reaction mixture contained Tris-HCl buffer (16 mM, pH 8.0), NADH (73 μM), NBT (50 μM) and various concentrations (4–64 mg/L) of APS (not added to the control group). After adding PMS solution (15 μM, the blank was excluded) to the mixture, the reaction mixture was incubated at 25 °C for 2 min, and the absorbance (A) at 560 nm was measured against blank samples. Decreased absorbance of the reaction mixture indicated increased superoxide anion scavenging activity. Vitamin C was used as positive control. The superoxide anion scavenging rate (SR%) was calculated according to the following equation:

$$\text{SR}\%=[(A_{\text{control}}-A_{\text{APS or Vitamin C}})/A_{\text{control}}]\times 100$$

### 3.8. Hydrogen Peroxide Scavenging Activity Assay

Hydrogen peroxide scavenging activity of APS and standards was assayed by the method of Zhao [31]. Aliquot of H_2_O_2_ (1.0 mL, 0.1mM) and 1.0 mL of various concentrations of APS were mixed, followed by 100 μL 3% ammonium molybdate, 10 mL H_2_SO_4_ (2 M) and 7.0 mL KI (1.8 M). The mixed solution was titrated with 5 mM Na_2_S_2_O_3_ until the yellow color disappeared. The percentage scavenging effect was calculated as Scavenging rate (SR%) =[(*V*_0_ − *V*_1_)/*V*_0_] × 100, where *V*_0_ was volume of Na_2_S_2_O_3_ solution used to titrate the control sample in the presence of hydrogen peroxide (without APS), *V*_1_ was the volume of Na_2_S_2_O_3_ solution used in the presence of APS.

### 3.9. Hydroxyl Radicals Scavenging Activity Assay

The Fenton reaction system of a final volume of 2 mL contained 0.75 mM FeSO_4_, 0.75 mM 1,10-phenanthroline, 0.8 mM H_2_O_2_, 150 mM PBS buffer and APS (4–64 mg/L) or BHT (0.30–2.4 mg/L) at different concentrations. BHT was used as positive control. Reaction was started by adding H_2_O_2_ and the tubes were incubated for 60 min in a water bath at 37 °C [57]. The absorbance of the Fe^2+^–phenanthroline complex was measured at 536 nm. •OH scavenging by APS was calculated according to the following equation: SR% = [(*A*_1_ − *A*_0_)/(*A*_2_ − *A*_0_)] × 100 [*A*_0_: Control; *A*_1_:APS or BHT; *A*_2_: Blank without drug and H_2_O_2_].

### 3.10. Determination on the Activities of CAT, SOD, GPx and Anti-hydroxyl Radical

Mice were killed via dislocation, and livers were rapidly removed, weighed and made into 1% homogenates with normal saline at 0 °C. Two milliliters of homogenate was centrifuged at 2000 × g for 5 min, 100 μL supernatant was added to 900 μL normal saline and mixed, 10 μL of which was used for determination of CAT, SOD, GPx and anti-hydroxyl radical activities by using commercial monitoring kits respectively, following the manufacturer’s protocol.

### 3.11. Statistical Analysis

Data were expressed as means ± S.D. of three replicates and the tables represent the average of replicate experiments. Statistical differences between groups were analyzed by one-way analysis of variance (ANOVA) followed by least significant difference (LSD) *post hoc* multiple comparisons test using the statistical software package SPSS 16.0 for Windows (SPSS Inc., Chicago, IL, USA). Results were considered statistically significant at the probability (*P*) values < 0.05 level.

## 4. Conclusions

The present study shows that APS inhibit mitochondrial injury and swelling and also had significant ROS scavenging effect. APS clearly inhibit the generation of TBARS in mitochondria, which indicates that it can significantly protect the body from LPO. The inhibition of LPO and mitochondrial swelling suggests that APS may inhibit the generation of ROS in mitochondria. APS can interfere with the oxidation process by reacting with free radicals, and also by acting as ROS scavengers. From the standpoint of health and longevity, our results indicate that APS may protect mitochondria from oxidative damage. Moreover, APS notably increase the activities of the antioxidases. In conclusion, APS would have a beneficial effect on protecting mitochondria while maintaining efficient cellular metabolism.

The electron transport chain (ETC) in the mitochondrial inner membrane is actively involved in adenosine triphosphate (ATP) synthesis in combination with respiration, which consumes approximately 90% of the oxygen uptake of the tissue cells. A fraction of the oxygen is incompletely reduced to generate ROS and organic free radicals, which are usually disposed of by the coordinated function of antioxidant enzymes. If ROS escape, they may facilitate LPO and cause oxidative damage, at least transiently, to the inner membrane. Iron-induced LPO alter mitochondrial respiration and oxidative phosphorylation (OXPHOS). The impaired ETC works less efficiently in ATP synthesis and generates more ROS, which will cause further oxidative damage to various biomolecules in mitochondria. In the aging process, oxidative damage ultimately leads to a progressive decline in bioenergetic function and enhanced mitochondrial oxidative stress. The energy depletion and enhanced oxidative stress can lead to the aging process. APS can protect mitochondria by scavenging ROS, inhibiting LPO and mitochondrial swelling, and increasing the activities of antioxidant enzymes. Therefore, perhaps APS ameliorate mitochondrial dysfunction ultimately by improving energy metabolism; this may be the hypothetical mechanism of APS on mitochondrial protection and anti-aging activity.

## Figures and Tables

**Table 1:** The effects of *Astragalus* polysaccharides (APS) on thiobarbituric acid reactive substances (TBARS) in liver and brain mitochondria (n = 6). Data were expressed as means ± S.D. and statistical differences between groups were analyzed by one-way analysis of variance (ANOVA).

Group	Concentration (mg/L)	C(liver) (nmol/mg protein)	Liver IR%	C(brain) (nmol/mg protein)	Brain IR%
**Normal**	—	0.62 ± 0.38 b		0.38 ± 0.29 b	
**Model**	—	4.32 ± 1.65		4.84 ± 1.62	
**APS**	2.0	3.56 ± 0.89	20.54	4.05 ± 1.16	17.71
	4.0	3.08 ± 0.53	33.51	3.46 ± 0.78	30.94
	8.0	2.37 ± 0.65 a	52.70	2.88 ± 0.66 a	43.95
	16.0	1.65 ± 0.49 b	72.16	1.96 ± 0.57 b	64.57
	32.0	0.92 ± 0.35 b	91.89	1.35 ± 0.79 b	78.25

a *P* < 0.05

b *P* < 0.01, compared with model group.

C: TBARS contents; IR: inhibition rate; APS: *Astragalus* polysaccharides.

**Table 2:** The effect of APS on liver mitochondrial permeability transition induced by Ca^2+^ (n = 6). Data were expressed as means ± S.D. and statistical differences between groups were analyzed by one-way analysis of variance (ANOVA).

*A*_540nm_						

Group	Normal	Model	RR (0.3 μM)	RR (0.5 μM)	APS (32 mg/L)	APS (64 mg/L)
**0 min**	0.481 ± 0.035	0.493 ± 0.033	0.486 ± 0.024	0.479 ± 0.038	0.480 ± 0.034	0.491 ± 0.026
**2 min**	0.452 ± 0.031 a	0.405 ± 0.023	0.429 ± 0.028	0.448 ± 0.029 a	0.422 ± 0.024	0.443 ± 0.022 a
**5 min**	0.431 ± 0.033 b	0.366 ± 0.026	0.402 ± 0.023 a	0.432 ± 0.025 b	0.394 ± 0.018	0.419 ± 0.023 b
**10 min**	0.413 ± 0.030 b	0.341 ± 0.033	0.383 ± 0.031 a	0.415 ± 0.037 b	0.376 ± 0.036	0.407 ± 0.035 b
**15 min**	0.398 ± 0.046 b	0.309 ± 0.034	0.376 ± 0.026 b	0.400 ± 0.035 b	0.361 ± 0.036 a	0.393 ± 0.036 b
**30 min**	0.369 ± 0.033 b	0.276 ± 0.041	0.355 ± 0.038 b	0.371 ± 0.043 b	0.336 ± 0.035 a	0.366 ± 0.045 b

a *P* < 0.05,

b *P* < 0.01, compared with model group.

A: Absorbance; RR: ruthenium red.

**Table 3:** The scavenging effect of APS on O_2^•^_^−^ and •OH (*n* = 6). Data were expressed as means ± S.D. and statistical differences between groups were analyzed by one-way analysis of variance (ANOVA).

Group	Conc. (mg/L)	*A*_560nm_(O_2^•^_^−^)	SR% (O_2^•^_^−^)	Group	Conc. (mg/L)	*A*_536 nm_ (•OH)	SR% (•OH)
**Control**	—	0.376 ± 0.038		**Blank**		0.136 ± 0.019 b	
**Vitamin C**	4	0.343 ± 0.042	8.78	**Control**		0.034 ± 0.016	
	8	0.271 ± 0.048 b	27.93	**BHT**	0.30	0.045 ± 0.020	10.78
	16	0.220 ± 0.033 b	41.49		0.60	0.072 ± 0.018 b	37.25
	32	0.117 ± 0.026 b	68.88		1.20	0.101 ± 0.028 b	65.69
	64	0.065 ± 0.028 b	82.71		2.40	0.128 ± 0.025 b	92.16
**APS**	4	0.361 ± 0.028	3.99	**APS**	4	0.042 ± 0.017	7.84
	8	0.335 ± 0.029	10.90		8	0.066 ± 0.026 a	31.37
	16	0.286 ± 0.037 b	23.94		16	0.088 ± 0.021 b	52.94
	32	0.220 ± 0.042 b	41.49		32	0.107 ± 0.013 b	71.57
	64	0.148 ± 0.036 b	60.64		64	0.121 ± 0.014 b	85.29

a *P* < 0.05,

b *P* < 0.01, compared with control group.

Conc: Concentration; BHT: butylated hydroxytoluene; SR: Scavenging rate.

**Table 4:** The scavenging effect of APS on H_2_O_2_ (*n* = 6). Data were expressed as means ± S.D. and statistical differences between groups were analyzed by one-way analysis of variance (ANOVA).

Group	Concentration (mg/L)	V (Na_2_S_2_O_3_ mL)	SR%
**Control**	—	1.638 ± 0.057	
**APS**	4	1.544 ± 0.051^a^	5.74
	8	1.364 ± 0.042^b^	16.73
	16	1.116 ± 0.029^b^	31.87
	32	0.860 ± 0.032^b^	47.50
	64	0.688 ± 0.026^b^	58.00
	128	0.536 ± 0.023^b^	67.28

a *P* < 0.05,

b *P* < 0.01, compared with control group.

V: Volume.

**Table 5:** Effects of APS on the CAT, SOD, GPx and anti-hydroxyl radical activities in mice liver *in vivo* (n =10). Data were expressed as means ± S.D. and statistical differences between groups were analyzed by one-way analysis of variance (ANOVA).

Group	Dose (mg/kg/d)	CAT (U/mg protein)	SOD (U/mg protein)	GPx (U/mg protein)	Anti-OH (U/mg protein)
**Normal**	—	14.3 ± 3.1 b	268 ± 45 b	58.1 ± 7.8 b	93.7 ± 15.2 b
**Model**	—	8.5 ± 3.0	203 ± 32	41.3 ± 9.3	62.6 ± 14.1
**Vit E**	100	12.6 ± 2.5 b	266 ± 28 b	51.7 ± 6.8 a	88.3 ± 10.3 b
**ASP 1**	100	9.2 ± 2.4	227 ± 36	45.3 ± 7.7	68.1 ± 8.6
**ASP 2**	200	11.4 ± 2.6 a	243 ± 29 b	53.5 ± 6.6 b	75.9 ± 10.8 a
**ASP 3**	300	14.1 ± 3.3 b	271 ± 33 b	57.3 ± 7.2 b	90.5 ± 13.5 b

a *P* < 0.05,

b *P* < 0.01, compared with model group.

CAT: catalase; SOD: surperoxide dismutase; GPx: glutathione peroxidase; Anti-•OH: anti-hydroxyl radical; Vit E: vitamin E.
